# Supplementary material for: Endothelial Autophagy in Coronary Microvascular Dysfunction and Cardiovascular Disease
Source: Cells. 2022 Jun 30;11(13):2081. doi: 10.3390/cells11132081 (PMC9265562; doi:10.3390/cells11132081)
Supplement: Supplementary file 1 [file cells-11-02081-s001.zip › cells-1763575-supplementary.pdf]

Table S1. Mechanistic Studies Implicating Endothelial Autophagy or Mitophagy in Cardiovascular Diseases.

Disease Model	Experimental Approach	Role of Autophagy/ Mitophagy	Mechanism	Reference
Obstructive CAD	*In vitro*, H2O2-induced oxidative stress in HCAECs;	Protective, pro-survival	MicroRNA-103 could protect HCAECs against H2O2-induced injury by preventing Bcl-2/ BNIP3-mediated suppression of end-stage autophagy	[105]
	Endogenous ROS-induced oxidative stress in Nox2-Tg^{EC} mice *in vivo and* in primary MHECs *in vitro:* *Ex vivo*, coronary microvessel relaxation studies;	Protective, pro-survival	Endogenous ROS oxidative stress protected MHECs from oxidant-induced cell death by increasing AMPK-mTOR-mediated-autophagy	[106]
	In vitro, 1% O_2-induced hypoxia in primary rat CMECs; *Ex vivo*, coronary microvessel relaxation studies; *In vivo*, MI model in SD rats;	deleterious, pro-apoptosis	FoxO3α-mediated autophagy aggravated hypoxia-induced rat CMECs dysfunction and apoptosis	[108]
	In vitro, 95% N_2 and 5% CO_2-induced hypoxia in HUVECs;	Protective, pro-survival	Rcan1-1L overexpression-induced mitophagy contributed to cell survival under hypoxic conditions	[109]
	In vitro, OGD/R injury in human CMECs;	Protective, pro-survival	Dexmedetomidine could protect human CMECs against OGD/R injury by activating PPARδ-AMPK-PGC-1α pathway-mediated autophagy	[110]
	In vitro, OGD injury in human CMECs; *In vivo*, MI model in Wistar rats;	deleterious, pro-apoptosis	PEP protected human CMECs against OGD-induced injury by inhibiting autophagy via promoting the mTOR pathway	[111]
	In vitro, ox-LDL, Ang-II, and HG-induced injuries in HCAECs;	Protective, pro-survival	Aspirin protected HCAECs against ox-LDL, Ang-II, and HG-induced injuries by inducing Beclin-1-dependent autophagy	[112]
	In vitro, H/R injury in human CMECs;	Protective, pro-survival	Tongxinluo protected human CMECs against H/R injury-induced apoptosis by promoting autophagy via activating the MEK/ERK pathway	[113]
	In vitro, H/R injury in primary rat CMECs; *In vivo*, I/R model in SD rats;	Protective, pro-survival	NAD+ protected CMECs from H/R or I/R injury by improving autophagy via activation and translocation of TFEB from the lysosomes to the nuclei	[114]
	In vitro, H/R injury in HUVECs;	Protective, pro-survival	Mitophagy activated by UA protected HUVECs against H/R injury by enhancing MQC, and thus improving cell viability and proliferation	[115]
	In vitro, H/R injury in primary MHECs; *In vivo*, I/R model in NR4A1 KO mice;	Protective, pro-survival	NR4A1 exacerbated heart microvascular I/R injury by inhibiting FUNDC1-dependent mitophagy and improving Mff-mediated mitochondrial fission by CK2α	[116]
	In vitro, H/R injury in primary MHECs; *In vivo*, I/R model in Ripk3 KO mice;	Protective, pro-survival	Ripk3 aggravated mitochondrial apoptosis by suppressing FUNDC1-mediated mitophagy in cardiac IR injury	[117]
	In vitro, H/R injury in human CMECs; *In vivo*, I/R model in Beclin-1 overexpression mice;	Protective, pro-survival	Beclin1 overexpression in CMECs exhibited reduced NLRP3 inflammasome activation and IL-1β production by promoting TNFAIP3 expression	[118]
	In vitro, H/R injury in human CMECs; *In vivo*, I/R model in Beclin-1 overexpression mice;	Protective, pro-survival	Beclin1 overexpression in CMECs inhibited caspase-4 inflammasome activation, suppressed pyroptosis, and reduced IL-1β production	[119]
	In vitro, H/R injury in primary mouse CMECs; *In vivo*, I/R model in SERCA overexpression mice;	Protective, pro-survival	SERCA overexpression protected CMECs against I/R injury by preserving MQC, including mitophagy, via suppressing calcium overload, inactivating XO, and reducing intracellular/mitochondrial ROS	[120]
	In vitro, HUVECs and primary CMECs from MI mice; *In vivo*, MI model in miR-92a cardiac-specific KO mice;	Protective, pro-survival	MiR-92a inhibition improved EC function by inducing EC autophagy	[121]
	In vitro, H/R injury in HCAECs;	deleterious, pro-apoptosis	GA protected CAEC against H/R-induced damage by reducing ROS accumulation and inhibiting autophagy/mitophagy	[122]

Table S1-continued

**Obstructive CAD**	*In vitro*, H/R injury in neonatal rat CMECs; *In vivo*, I/R model in SD rats;	deleterious, pro-apoptosis	Melatonin protected CMECs against I/R injury by suppressing autophagy via the AMPK/mTOR pathway	[123]
	In vitro, H/R injury in primary mouse CMECs; *In vivo*, I/R model in AMPKα KO mice;	deleterious, pro-apoptosis	Melatonin protected CMECs against I/R injury by inhibiting mitophagy-mediated cell death via the Drp1-VDAC1-HK2-mPTP-PINK1/Parkin axis in an AMPKα-dependent manner	[124]
	In vitro, H/R injury in HCAECs; *Ex vivo*, EPR Spectrometry (Quantitation of NO); *In vivo*, I/R model in ErbB2 EC-KO mice;	deleterious, pro-apoptosis	Nrg1β protected cardiac ECs against I/R injury by preventing ATG5-required-autophagy-induced Trx2 degradation and rescuing eNOS function via upregulating ErbB2	[125]
	In vitro, H/R injury in primary rat CMECs; exosomes from hUCMSCs; *In vivo*, I/R model in SD rats following exosomes injection;	deleterious, pro-apoptosis	LncRNA UCA1 transferred from hUCMSCs-derived exosomes protected CMECs against H/R injury by inhibiting autophagy through damaging miR-143-mediated degradation of Bcl-2	[126]
**Obstructive CAD (PCI-Associated ISR)**	*In vitro*, Sirolimus or Paclitaxel treatment in HAECs; *Ex vivo*, re-endothelialization assay, aortic ring assay, and imaging of autophagic endothelium;	Protective, pro-apoptosis	EC autophagy induced by sirolimus and paclitaxel by regulating LC3B, Bcl-2, and p53 was beneficial to the human aorta in suppressing reendothelialization and revascularization, as well as NO production	[130]
	In vitro, EGCG treatment (131) in HUVECs or HUVECs were seeded on surfaces of different steel substrates (132);	Protective, pro-apoptosis	EGCG and 316L SS were reported to inhibit HUVECs proliferation by upregulating autophagic genes Atg 5/7/12 and cell apoptosis genes caspase 3/8/9 and Fas to suppress the occurrence of ISR	[131,132]
	In vitro, Rapamycin treatment in HUVECs, and HUVECs /platelets co-culture; *In vivo*, deep vein thrombosis model in rats;	deleterious, pro-EC membrane remodeling	Autophagy-dependent endothelial membrane ruffling and platelet-EC adhesion enhancement were crucial for rapamycin-eluting stent-induced stent thrombosis	[133]
**HFpEF, Angiogenesis**	*In vitro*, VEGF-A treatment in HUVECs; *In vivo*, MI model in mice;	Protective, pro-angiogenesis	VEGF-A improved angiogenesis after MI by promoting ROS production and increasing ER stress-induced autophagy	[151]
	In vitro, 1% O_2-induced hypoxia in MHECs, and HUVECs; *Ex vivo*, Mouse Aortic Ring Assay; *In vivo*, MI model in AGGF1 KO, Beclin1 KO, and ATG5 KO mice;	Protective, pro-angiogenesis	AGGF1 KO mice showed suppressed angiogenesis by disturbing the initiation of autophagy via reducing the assembly of Beclin1-Vps34-Atg14 complex	[93]
	In vitro, human LepR siRNA treatment in human CMECs, HUVECs, and primary ECs from mouse hearts and lungs; *In vivo*, TAC model in LepR EC-KO mice, and the endomyocardial biopsy from HF patients;	Protective, pro-angiogenesis	EC LepR deletion proved to promote cardiac angiogenesis by enhancing autophagosome formation via suppressing Akt/mTOR signaling	[152]
	In vitro, Ang II treatment in primary mouse CMECs; *Ex vivo*, aortic sprout assay; *In vivo*, Ang II infusion in SIRT3 KO and SIRT3-Tg^{EC} mice;	Protective, pro-angiogenesis	Sirt3 deletion was found to aggravate Ang II-induced PINK1/Parkin acetylation aberrance, resulting in impaired mitophagy, excessive mtROS generation, and damaged angiogenic capacity of CMECs	[153]
	In vitro, ECs from SFVs of CAD patients and HUVECs; *In vivo*, Matrigel plug assay in CD-1 mice, and histological analysis in TCHP KO mice;	Protective, pro-angiogenesis	The TCHP deletion impaired autophagosome maturation, accumulated p62 in the heart and cardiac vessels, and damaged cardiac vascularization	[154]
	In vitro, VEGF treatment in HUVECs, HCAECs, and MAECs *Ex vivo*, mouse aortic ring assay; *In vivo*, Matrigel plug assay, and hindlimb ischemia model in TFEB EC-KO mice and TFEB-Tg^{EC} mice;	Protective, pro-angiogenesis	TFEB improved angiogenesis through activation of AMPKα and autophagy via TFEB-dependent transcriptional upregulation of MCOLN1	[155]
	In vitro, H/R injury in primary mouse CMECs; *In vivo*, I/R model in C57BL/6 mice following sRAGE treatment;	deleterious, anti-angiogenesis	soluble RAGE exerted increased angiogenesis and reduced apoptosis through inhibition of autophagy by activating signal transducer and activator of transcription 3 (STAT3) pathway	[157,158]

Table S1-continued

HFpEF, Angiogenesis	*In vitro,* MGO treatment in BAECs and HUVECs;<br>*Ex vivo,* mouse aortic ring assay;<br>*In vivo,* C57BL/6J and Akita mice with mTempol treatment and DB/DB diabetic mice;	deleterious,<br>anti-angiogenesis	MGO exhibited reduced endothelial angiogenesis through RAGE-required, peroxynitrite (ONOO)–mediated, and autophagy-triggered VEGFR2 degradation	[156]
	In vitro, CPCD treatment in MEECs, HMEC-1, and primary mouse MAECs;<br>*In vivo,* CPCD treatment in zebrafish embryonic;	anti-angiogenesis	CPCD inhibited sprouting angiogenesis during zebrafish embryonic development by enhancing autophagy in ECs via inhibition of VEGFR2/AKT pathway	[159]
	SiNPs treatment in HUVECs *in vitro* and in ICR mice *in vivo*;	deleterious,<br>anti-angiogenesis	SiNPs could enhance autophagic activity, disturb EC homeostasis, and impair angiogenesis (via inhibitory effect on ICAM-1 and VCAM-1 expression)	[160]
HFpEF, Fibrosis	*In vitro,* MoS2 QDs treatment in HUVECs and HCAECs;	Protective,<br>anti-EndMT,<br>pro-angiogenesis	Restoring TFEB-mediated autophagic flux by MoS2 QDs could inhibit TGF-β-mediated EndMT and promote angiogenesis in HCAECs by triggering TFEB nucleus translocation	[166]
	IL6 neutralizing antibody treatment in HMVECs *in vitro* and in ATG5 EC-KO mice *in vivo;*	Protective,<br>anti-EndMT,	The disruption of autophagy in ECs could induce pathological IL6-dependent EndMT and aggravate heart fibrosis	[167]
	In vitro, 1% O2-induced hypoxia in human CMECs;	Protective,<br>anti-EndMT,<br>pro-angiogenesis	Upregulation of autophagy could prevent hypoxia-induced EndMT, cell apoptosis, and enhance angiogenesis by inhibiting the NF-κB-Snail pathway in human CMECs	[149,150]
	In vitro, DOX and/or Irisin treatment in primary in CMs and CFs from neonatal mice and CMECs from adult mice;<br>*In vivo,* DOX and/or Irisin treatment in C57BL6 mice;	Protective,<br>anti-EndMT,	Irisin alleviated DOX-dependent cardiac perivascular fibrosis by restraining EndMT by restoring autophagy in ECs, resulting in reduced ROS accumulation and inhibited NF-κB-Snail pathway	[168]
	In vitro, AGEs treatment in HUVECs;<br>*In vivo,* TAC model in RAGE KO mice;	deleterious,<br>pro-EndMT,	The suppression of autophagy by RAGE inactivation mitigated TAC-induced cardiac fibrosis through repressing EndMT	[169]
	In vitro, H2O2 + rapamycin treatment in HCAECs;	deleterious,<br>pro-EndMT,	Inducing autophagy by rapamycin promoted H2O2-induced EndMT through activating the TGF-β pathway	[170]
DCM	*Ex vivo* organ culture model;	No description	Prolonged exposure of fetal mouse heart to sucrose or mannitol could induce severe lysosomal derangements and prominent autophagy in ECs	[181]
	In vitro, Mst1-enriched exosomes isolated from HG-treated primary mouse CMECs cocultured with HG-treated CMs;<br>*In vivo,* STZ-induced type 1 DM model in Mst1-Tg^{EC} mice;	Protective,<br>pro-survival	Mst1-enriched exosomes (transferred from CMECs to CMs) mediated the inhibition of Beclin1/Bcl2-dependent autophagy and enhancement of Bcl-2/Bax-associated apoptosis in CMs	[184]
	In vitro, HG-treatment in primary mouse CMECs;<br>*In vivo,* STZ-induced type 1 DM model in Mst1 KO and Mst1 transgenic mice;	Protective,<br>pro-survival	Mst1 contributed to CMD in DM by directly inhibiting autophagy and inducing apoptosis in CMECs	[185]
	In vitro, HG-treatment in primary rat CMECs;	Protective,<br>pro-survival	Upregulation of autophagy was reported to rescue HG-induced EC apoptosis through the Akt-mTOR signal pathway	[186]
	In vitro, HG/PA or PA treatment in HAECs or RAECs;<br>*In vivo,* STZ-induced type 1 DM and HFD-induced type 2 DM model in C57BL/6 mice (186);	Protective,<br>pro-survival	Mitophagy was shown to protect mitochondrial integrity and prevent HG/PA-induced EC apoptosis via the PINK1-Parkin pathway and retard HG/PA-induced EC senescence via the AMPK pathway	[187,188,189]
	In vitro, ox-LDL treatment in HUVECs;	Protective,<br>pro-survival	Improving Bnip3-dependent mitophagy could rescue ox-LDL-induced EC damage, resulting in restored activation of mitochondrial respiration complexes , reduced ROS production, and increased EC viability	[190]

Table S1-continued

DCM	*In vitro,* HG and/or metformin treatment in HUVECs; *Ex vivo,* mouse aortic ring assay; *In vivo,* ATG7 EC-KO mice, ATG7 retinal vessels silence mouse, and DB/DB mice following metformin treatment;	deleterious, pro-apoptosis	Downregulation of autophagy relieved HG-induced endothelial impairment via the GLI1 dependent-Hedgehog pathway	[191]
	In vitro, siATG7 treatment in HUVECs; *Ex vivo,* aerobic heart perfusion and I/R models in hearts of ATG7 EC-KO mice;	Protective, pro-fatty acid oxidation	Downregulation of EC autophagy by ATG7 KO impaired cardiac fatty acid stores and repressed the reliance of hearts on fatty acid oxidation as the primary fuel source both upon insulin insult and during I/R injury	[193]
	In vitro, TNF-α and/or PA treatment in primary adult rat CMECs and CMs;	deleterious, pro-insulin resistance	TNF-α-induced EC autophagy, cooperating with the NF-κB signaling, resulted in upregulated FATP4 expression in CMECs, facilitated CMEC PA transcytosis, and aggravated insulin resistance	[194]
Others/KD	*In vitro,* co-culture of PBMCs from acute KD patients with HCAECs;	deleterious, pro-inflammation	The PBMCs collected from KD patients with fever could promote HCAECs secreting chemokines and pro-inflammatory factors via inducing autophagy	[197]
	In vivo, ginsenoside Rb1 treatment in 10% BSA IP injection-induced mouse KD model.	Protective, anti-inflammation	Ginsenoside Rb1 could effectively alleviate coronary artery lesion in a mouse KD model, possibly by upregulating the AMPK/mTOR/P70S6 pathway-mediated autophagy to prevent EC injury	[198]
	In vitro, TNF-α and/or Resveratrol treatment in HCAECs;	Protective, anti-inflammation	Activation of autophagy contributed to the anti-inflammatory effects of Resveratrol in TNF-α-treated HCAECs	[199]
Others/Heart Regeneration	*In vivo,* cryoinjury of the heart in zebrafish following metformin treatment;	Protective, pro-regeneration	Autophagy was positively correlated with the metformin-mediated cardiac regeneration acceleration in zebrafish, including epicardial, endocardial, and vascular endothelial regeneration	[200]

Abbreviations: CAD, coronary artery disease; HCAECs, human coronary artery endothelial cells (ECs); Nox2, NADPH oxidases 2; ROS, reactive oxygen species ROS; MHECs, mouse heart ECs; Tg^{EC}, EC-specific overexpression; AMPK, AMP-activated protein kinase; foxO3α, Forkhead box O3 alpha; CMEC, cardiac microvascular ECs; SD, Sprague–Dawley; MI, myocardial infarction; HUVECs, human umbilical vein ECs; Rcan1-1L, the regulator of calcineurin 1-1L; OGD/R, oxygen-glucose deprivation and re-oxygenation; PPARs, the peroxisome proliferator-activated receptors; PGC-1α, peroxisome proliferator-activated receptor gamma coactivator 1-alpha; PEP, polysaccharides from Enteromorpha prolifera; Ang II, angiotensin II; HG, high glucose; H/R, hypoxia/reoxygenation; MEK, mitogen-activated protein kinase; ERK, extracellular signal-regulated kinase; I/R, ischemia/reperfusion; TFEB, transcription factor EB; UA, urolithin A; MQC, mitochondrial quality control; NR4A1, nuclear receptor subfamily 4 group A member 1; KO, knockout; FUNDC1, FUN14 domain-containing protein 1; Mff, mitochondrial fission factor; CK2α, serine/threonine kinase casein kinase2 α; Ripk3, receptor-interacting protein kinase 3; NLRP3, NACHT, LRR, and PYD domains-containing protein 3; TNFAIP3, tumor necrosis factor-alpha-induced protein 3; SERCA, sarcoplasmic/endoplasmic reticulum Ca2+-ATPase; XO, xanthine oxidase; GA, Glycyrrhizic acid; Drp1, dynamin-related protein 1; VDAC1, voltage-dependent anion channel 1; HK2, hexokinase 2; mPTP, mitochondrial permeability transition pore; PINK1, phosphatase and tensin homolog (PTEN)-induced putative kinase 1; Nrg1β, neuregulin 1β; ErbB2, Erb-B2 receptor tyrosine kinase 2; ATG, autophagy-related gene; Trx2, thioredoxin; eNOS, endothelial nitric oxide synthase; hUCMSC, human umbilical cord mesenchymal stem cell; LncRNA, long noncoding RNA; HAECs, human aortic ECs; PCI, percutaneous coronary intervention; ISR, in-stent restenosis; EGCG, epigallocatechin-3-gallate; 316L SS, nickel-containing austenitic 316L stainless steel; TAC, transverse aortic constriction; LepR, leptin receptors; HF, heart failure; AGGF1, Angiogenic Factor With GPatch And FHA Domains 1; Sirt3, Sirtuin 3; SFVs, superficial forearm veins; TCHP, Trichoplein; TFEB, transcription factor EB; MCOLN1, mucolipin-1; MAECs, mouse aorta endothelial cells; MGO, methylglyoxal; RAGE, the receptor for advanced glycation end products; BAECs, bovine aortic endothelial cells; CPCD, Capsicodendrin; MEECs, mouse embryonic endothelial cell line; HMEC-1, human microvascular endothelial cell line; SiNPs, Silica nanoparticles; EndMT, endothelial-mesenchymal transition; MoS2 QDs, Molybdenum disulfide quantum dots; TGF-β, transforming growth factor β; IL6, interleukin 6; HMVECs, human dermal microvascular endothelial cells; CMs, cardiomyocytes; CFs, cardiac fibroblasts; DOX, doxorubicin; AGEs, advanced glycation end products; DCM, diabetic cardiomyopathy; Mst1, mammalian sterile 20-like kinase 1; STZ, streptozotocin; DM, diabetes mellitus; HG/PA, HG and palmitate acid; RAECs, rat aortic endothelial cells; GLI1, Glioma-associated oncogene homolog 1; FATP4, fatty acid transporter protein 4; PBMCs, peripheral blood mononuclear cells; KD, Kawasaki disease; BSA, bovine serum albumin; IP, intraperitoneal;
